# Pupil drift rate indexes groove ratings

**DOI:** 10.1038/s41598-022-15763-w

**Published:** 2022-07-08

**Authors:** Connor Spiech, George Sioros, Tor Endestad, Anne Danielsen, Bruno Laeng

**Affiliations:** 1 RITMO Centre for Interdisciplinary Studies in Rhythm, Time and Motion, University of Oslo, Postboks 1133 Blindern, 0318 Oslo, Norway; 2 Department of Psychology, University of Oslo, Oslo, Norway; 3 Department of Musicology, University of Oslo, Oslo, Norway

**Keywords:** Perception, Human behaviour, Auditory system, Attention, Visual system

## Abstract

Groove, understood as an enjoyable compulsion to move to musical rhythms, typically varies along an inverted U-curve with increasing rhythmic complexity (e.g., syncopation, pickups). Predictive coding accounts posit that moderate complexity drives us to move to reduce sensory prediction errors and model the temporal structure. While musicologists generally distinguish the effects of pickups (anacruses) and syncopations, their difference remains unexplored in groove. We used pupillometry as an index to noradrenergic arousal while subjects listened to and rated drumbeats varying in rhythmic complexity. We replicated the inverted U-shaped relationship between rhythmic complexity and groove and showed this is modulated by musical ability, based on a psychoacoustic beat perception test. The pupil drift rates suggest that groovier rhythms hold attention longer than ones rated less groovy. Moreover, we found complementary effects of syncopations and pickups on groove ratings and pupil size, respectively, discovering a distinct predictive process related to pickups. We suggest that the brain deploys attention to pickups to sharpen subsequent strong beats, augmenting the predictive scaffolding’s focus on beats that reduce syncopations’ prediction errors. This interpretation is in accordance with groove envisioned as an embodied resolution of precision-weighted prediction error.

## Introduction

### Theoretical background

The peculiar ability for music to enjoyably compel us to move in synchrony with its rhythm has generated considerable academic interest over the years^1–3^. This enjoyable urge to move (hereafter referred to as “groove” for simplicity’s sake) to music seems to be linked, at least in part, to the rhythm’s complexity^4–7^. Recently, this has been framed within predictive coding models of the mind, positing that groove-induced movements help to resolve sensory ambiguity regarding musical pulse and meter, thus minimizing prediction errors stemming from structural deviations like syncopation^8,9^. This is proposed to occur along an inverted U-shaped curve^10^. At low levels of complexity, there is little prediction error to resolve so movement isn’t needed to reinforce our metric model since it is already closely aligned with the rhythm. At more moderate levels of complexity, the body can be moved in synchrony with the basic beats of the groove, allowing proprioceptive inputs to reinforce the perceived pulse and meter of the rhythm and thus eradicate the sensory prediction errors. (Pulse here refers to the tempo in which you would tap your feet to the music, and meter to the way in which you would group these beats). In a more phenomenological approach, this body movement has been suggested to result in “participatory pleasure” by filling in the expected beat^11^. However, in highly complex rhythms, meter may become unclear, the prediction about the timing of notes may be weakened, and synchronous movements hindered^12^. Therefore, the greatest ‘precision’ in prediction errors (i.e., the most “predictable” prediction errors) occurs at moderate levels of metric complexity where these errors can be corrected by moving in a process of active inference^8^.

The above theory is also compatible with dynamic attending theory (DAT) accounts^13^ where active sensing (using movement to change sensory inputs) can entrain neural oscillations to relevant parts of the rhythm, selectively enhancing or suppressing their processing with attention^14,15^. While the elegance and plausibility of this account is enticing, strong evidence mapping behavior (i.e., the experience of groove) to neurophysiological processes using musically-relevant stimuli has remained elusive.

### Pupillometry of groove

If predictive coding underlies the enjoyable urge to move in response to music, then some neurophysiological marker of precision-weighted prediction errors should be found alongside the experience. One likely candidate is the neurotransmitter norepinephrine which has been hypothesized to encode the reliability (i.e., precision) of sensory predictions and enhance the signal-to-noise ratio of incoming information^16^. Pupillometry offers a convenient way to investigate this given its tight correlation to locus coeruleus activity, the brain’s primary norepinephrine producer^17–19^. Consequently, the pupil dilation response’s association with cognitive effort and attention allocation is well-documented^20–22^ and previous research shows that the pupil can index the deployment of attentional resources^23–27^. Since actively modulating the precision of prediction errors is likely to require attention^28^, it stands to reason that this process—if it is crucial to the experience of groove—could be observed using pupillometry. Initial findings are encouraging. Bowling, Graf Ancochea, Hove, and Fitch recorded greater pupil dilations in response to syncopated (and groovy) rhythms compared to unsyncopated (and less groovy) rhythms^29^ while Skaansar, Laeng, and Danielsen found that larger microtiming asynchronies elicited greater pupil dilations^30^. Thus, the question does not seem to be *whether* noradrenergic arousal is related to groove, but rather *how* it is related to groove and if this is consistent with the existing theories.

### The present study

We hypothesized that if the experience of groove is associated with an active process of suppressing prediction errors, then it should be reflected in stronger pupillometric arousal at moderate levels of syncopation where precision-weighted prediction error is highest and active inference is needed (and able) to correct it. To accomplish this, we decided to record participants’ pupil responses while listening to drumbeats varying in the amount of deviations from isochrony (and thus predictability). They also rated the drumbeats in terms of how much they wanted to move, how much they enjoyed them, and how energetic they were. Unique to our study, we characterized the deviations from isochrony in two orthogonal ways to investigate groove: events on unstressed or weak beats followed by subsequent strong beat events (pickups) and events on unstressed beats *not* followed by subsequent strong beat events (syncopations). The standard musicological definitions of pickups (also called anacruses or upbeats) and syncopation (see definitions in Refs.^31,32^) indicate that each deviation type has a different musical function: (a) pickups cue the following strong beat event and then fulfill it; (b) syncopations break this bond by omitting the strong beat event^32,33^. In other words, or psychological terms, pickups could be analogous to a priming stimulus prior to a temporal event while a syncopation seems more akin to its omission. Such a role of pickups in the experience of groove has been previously hypothesized^5,6^ although it was not investigated independently of syncopation. We predicted that pickups would elicit weaker pupil dilations than syncopations because syncopations lack a subsequent strong beat, making them more surprising and requiring more cognitive resources to suppress. For the purposes of this paper, we treat both pickups and syncopation as deviations that increase the rhythmic complexity of our stimuli (relative to rhythms without weak beat events) even if they may do so in different ways.

Many studies in cognitive psychology have employed simple drumbeats (e.g., kick-snare-hihat) to investigate rhythmic properties’ relation to groove^4,7,29,34–36^. However, these foundational studies tended to be more exploratory in nature and so several factors and parameters were uncontrolled, like event order, metric levels, perceived musicality, and the potential effect of pickups. To ensure that rhythmic complexity is the driving factor behind groove, the order of rhythmic events needs to be consistent in each condition since a kick-snare and snare-kick sound are qualitatively different and could therefore impact the urge to move. Moreover, the syncopated drumbeats in these studies tend to rely more heavily on faster metric levels whereas their lower or unsyncopated counterparts tend to remain at slower subdivisions, that is, subdivisions that are one level higher in the metric hierarchy. If these variations are systematic, they may introduce additional cognitive demands (e.g., attending to another metrical level) that scale in parallel to the amount of syncopation. Another pitfall is that stimuli sound nonmusical or cease to sound musical after being subjected to rigorous manipulation. If certain rhythmic conditions systematically sound less musical than others, this could affect the experience of groove and create or exaggerate differences that could then falsely be attributed to metric complexity; indeed, experienced familiarity with the music has been shown to play a role in groove^34^. Finally, while prior groove studies rigorously accounted for syncopations, none explicitly examined the predictive role of pickups and its effect on groove.

Because individual differences in beat perception could affect the way subjects model the rhythms, and therefore their experience of groove, we also administered the Computerised Adaptive Beat Alignment Test (CA-BAT)^37,38^. With this information, we hoped to extend previous findings that demonstrated a more prominent inverted U-shaped relationship between groove and syncopation in musicians^39^ by directly probing beat perception abilities that have been linked to synchronizing to high- and low-groove music^40^, without necessarily identifying such ability with musicianship. Specifically, we expected to find divergent results between high and low beat perception performance at the upper end of rhythmic complexity. Since good beat perception would be necessary to generate a predictive model of the most complex repeating drumbeat, this should result in greater groove ratings for high performers on the CA-BAT but not the low performers.

## Methods

### Participants

We recruited 30 participants (seven women) with varying degrees of musical experience and expertise as assessed by a custom-made questionnaire and the CA-BAT. All participants provided informed consent in accordance with the Declaration of Helsinki^41^ and were compensated with a 100 NOK (~ €10) gift card. Ethical approval was granted by the Department of Psychology’s internal research ethics committee at the University of Oslo (reference number 8131575). The average age of our sample was 26.8 (range 18–42, SD 5.07 years) and the average time spent listening to music was 24.03 h per week (range 1–84, median 21). Eleven of our subjects reported no musical training while the remaining 19 had trained for an average of 8.47 years (range 1–20, SD 6.22 years). Of these, 11 of 19 subjects played multiple instruments, with eight playing stringed instruments, four percussion, two brass instruments, seven piano, one voice, and five other/electronic instruments for an average of 5 h per week (range 0–27, standard deviation 6.59 h). A summary of each subject’s demographics and performance can be found in Table 1 of the Supplementary Materials.

### Stimuli

To ensure that our behavioral and pupillometry results reflected rhythmic complexity, each drumbeat followed the same order of events (alternating kicks and snare hits over a zeitgeber hihat, with an extra kick in the second bar) at the same metric level (that of the quaver) using the algorithm proposed by Sioros et al.^32^. Furthermore, we designed each stimulus with musicality in mind, starting with a standard back-beat rock drumbeat. It should be stressed here that our operationalization of Rhythmic Complexity narrowly treats any deviation from isochrony as an increase in complexity. While differing somewhat from the previously used Syncopation Index^7^ in that it distinguishes between pickups and syncopations and avoids assigning scalar weights, it orders our stimuli in the same manner. How this maps onto psychological perceptions of complexity is an open question.

**Figure 1:**
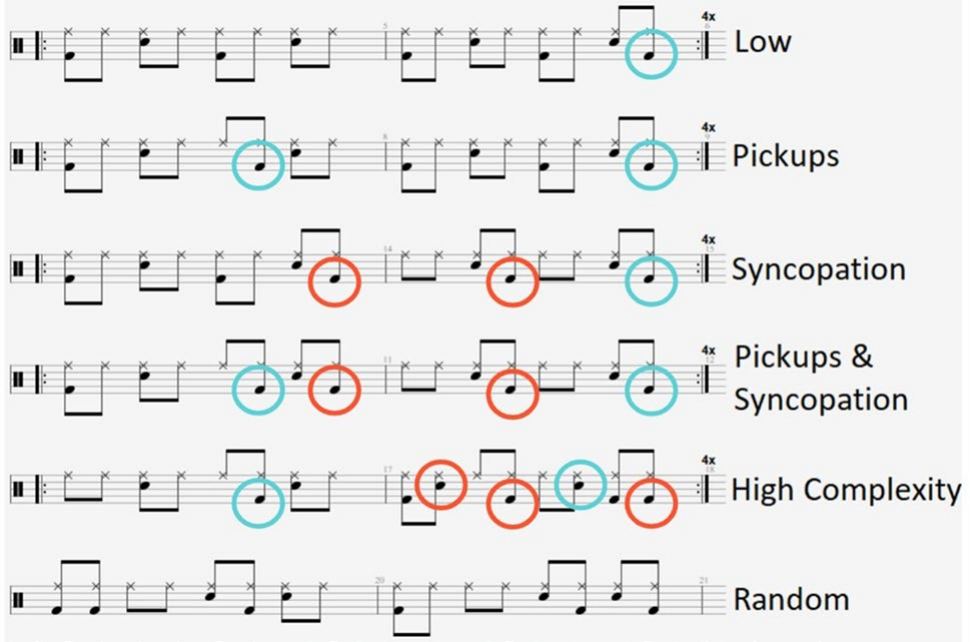
Musical notation for our drumbeat stimuli. Pickups are circled in blue while syncopations are circled in orange.

We settled on six different drumbeats: (1) a low complexity pattern with no pickups or syncopations (Low), (2) a pattern made moderately complex with pickups (Pickups), (3) a pattern made moderately complex with syncopation (Syncopation), (4) a pattern made moderately complex with both pickups and syncopation (Pickups and Syncopation), (5) a pattern made highly complex with more pickups and syncopations (High Complexity), and (6) a random condition where the event placements were pseudorandom (meeting our control criteria) and did not loop (Random). Except for the random condition, each drumbeat consisted of four two-bar patterns at 100 beats per minute for a total duration of 19.2 s. At the end of each two-bar pattern a kick drum stroke on the last eighth note position, that is, a pick-up to the first beat in bar 1, signals the start/end of a new pattern. The “random” condition was different from the others in that the pattern was randomly generated and varied with each repetition. Notations of each drumbeat are presented in Fig. 1 and sound files can be found here: https://osf.io/sd5up/?view_only=fa6bd354eb214368b77da9d5f18abcf1.

All stimuli were composed in Ableton Live, using MaxForLive devices for the automation of transformations, and produced in Reaper and then edited to appropriate lengths with Audacity^42^.

### Procedure

Pupil diameters were continuously sampled at 60 Hz using a SensoMotoric Instruments (SMI) RED250 eye tracker mounted beneath a 22-inch LED monitor in a dimly lit room situated 70 cm away from the subject. After a five-point (arranged in a cross) calibration and validation procedure, participants were instructed to passively listen to each drumbeat and immediately after rate each by how much they felt compelled to move (“I did not want to move at all” vs. “I wanted to move a lot” with movement being specified to include tapping or nodding), how much they liked the drumbeat (“I did not enjoy it at all” vs. “I enjoyed it a lot”), and how energetic the drumbeat sounded (“The drumbeat was very calm” vs. “The drumbeat was very excited”) using visual analogue scales that spanned half the width of the monitor with each key press corresponding to a jump of seven pixels. This scale granularity was not visible to the subject and the sensitivity was titrated to feel natural during piloting. The first two questions were used to measure groove while the last acted as a catch question and to control for perceived energetic arousal of the stimuli. During each trial, subjects fixated on a black fixation cross presented on a gray background generated with Psychtoolbox-3 for MATLAB^43^. The first three seconds of fixation were silent, serving as a baseline. Subsequently, a stereo drumbeat stimulus was played at a comfortable volume from two Genelec speakers (model 8030 W) flanking the screen with a subwoofer beneath the desk to enhance the bass since previous research suggests that it plays an important role in groove and establishing the beat for sensorimotor synchronization^44–47^. Each stimulus was presented ten times in a pseudorandom order such that no stimulus could repeat back to back. Thus, all subjects completed 60 trials and were permitted to take self-paced breaks every five trials. After the main portion of the experiment, each participant then completed the CA-BAT with the entire experiment lasting about one hour.

### Behavioral analysis

All subjects’ ratings of Urge to Move, Enjoyment, and Perceived Arousal were z-scored to control for individual differences in the way that subjects used the visual analog scales. The z-scored ratings of each trial were averaged for each drumbeat for each subject and then summary statistics were calculated at the group level for each drumbeat. To investigate beat perception, we grouped participants into High (N = 15) and Low (N = 15) Performance using a median split on their ability scores from the CA-BAT. The distribution of Beat Perception Ability scores as well as its significant correlations with our demographics measures are plotted in Supplementary Figs. 1–3. Differences between the High Complexity and Random drumbeats were compared using a mixed analysis of variance (ANOVA) with Beat Perception Performance group (High or Low) as a between-subjects factor and Rhythmic Complexity (High Complexity or Random) as a within-subject factor.

To replicate past findings of an inverted-U relationship between rhythmic complexity and groove, we fit mixed effects models to our subjects’ ratings (Urge to Move, Enjoyment, Perceived Arousal). In keeping with standard practice, we first fit intercepts-only models with random effects of Subject and Stimulus Repetition and compared them to models with Rhythmic Complexity as linear slopes (linear model) as well as linear and quadratic slopes (quadratic model). Model comparison was conducted via likelihood ratio tests and both the Akaike (AIC) and Bayesian information criterions (BIC)^48^. Follow-up t-tests using Satterthwaite’s method were carried out for best-fitting significant models.

To explore the possibly different effects of pickups and syncopation on perceived groove, we also organized our first four rhythms in a 2 × 2 design for a repeated measures ANOVA with factors Pickups (Present or Absent) vs. Syncopation (Present or Absent). The High Complexity and Random patterns were excluded from this analysis because they would unbalance the design. All behavioral plots and analyses were carried out using custom scripts in R (version 3.6.0^49^) and functions from the “dplyr”, “readr”, “ggplot2”, “lme4”, “lmerTest”, “effsize”, and “ez” packages.

### Pupillometry analysis

Data were exported using SMI BeGaze™ to a format suitable for preprocessing and analysis using custom scripts in R (version 3.6.0^49^) as well as functions from the “pupillometry” package^50^. First, the pupil time series for the right eye were locked to the stimuli onsets. Blinks were removed along with the preceding and succeeding 100 ms to eliminate edge artifacts resulting from partial occlusions of the pupil. Each trial was then smoothed using a 500 ms Hann window at 60 Hz and gaps smaller than 750 ms were interpolated with cubic splining. Next, the median pupil value from the last 1000 ms of each trial’s baseline period was subtracted from the rest of its time series to correct for random trial-to-trial fluctuations in a way that is less contaminated by noise than divisive baseline correction^51^. Finally, trials with more than 33% missing data were excluded and the remaining data was averaged in 100 ms bins for plotting and statistical analysis with the packages “ggplot2” and “ez”, respectively. Overall, this left us with 96.94% of valid pupil samples for analysis.

In addition to the pupil traces for individual trials, we were also interested in the rate at which these traces decayed since they could represent “decreasing attentional engagement”^22^. This is of particular importance to us because if norepinephrine is involved with suppressing precision-weighted prediction errors, then its firing would be more sustained while listening to groovier rhythms. Conversely, attention would disengage more rapidly to both simpler rhythms (which do not produce many prediction errors to suppress) and more complex rhythms (where prediction errors cannot be suppressed). Thus, for each of the four stimuli repetitions within a trial, we calculated the slope between the average pupil size in the first and last beats (300 ms) and took this pupil drift rate to represent attentional maintenance (at higher values) or fatigue (at lower values).

Finally, the same repeated measures ANOVA with Pickups (Present or Absent) and Syncopations (Present or Absent) as factors was computed with average Pupil Size as the dependent measure. Significant effects were then localized to time windows corresponding to the rhythmic manipulations of interest (i.e., the moments surrounding the pickups or the syncopations) by repeating the test in those windows.

## Results

### Behavioral results

**Figure 2:**
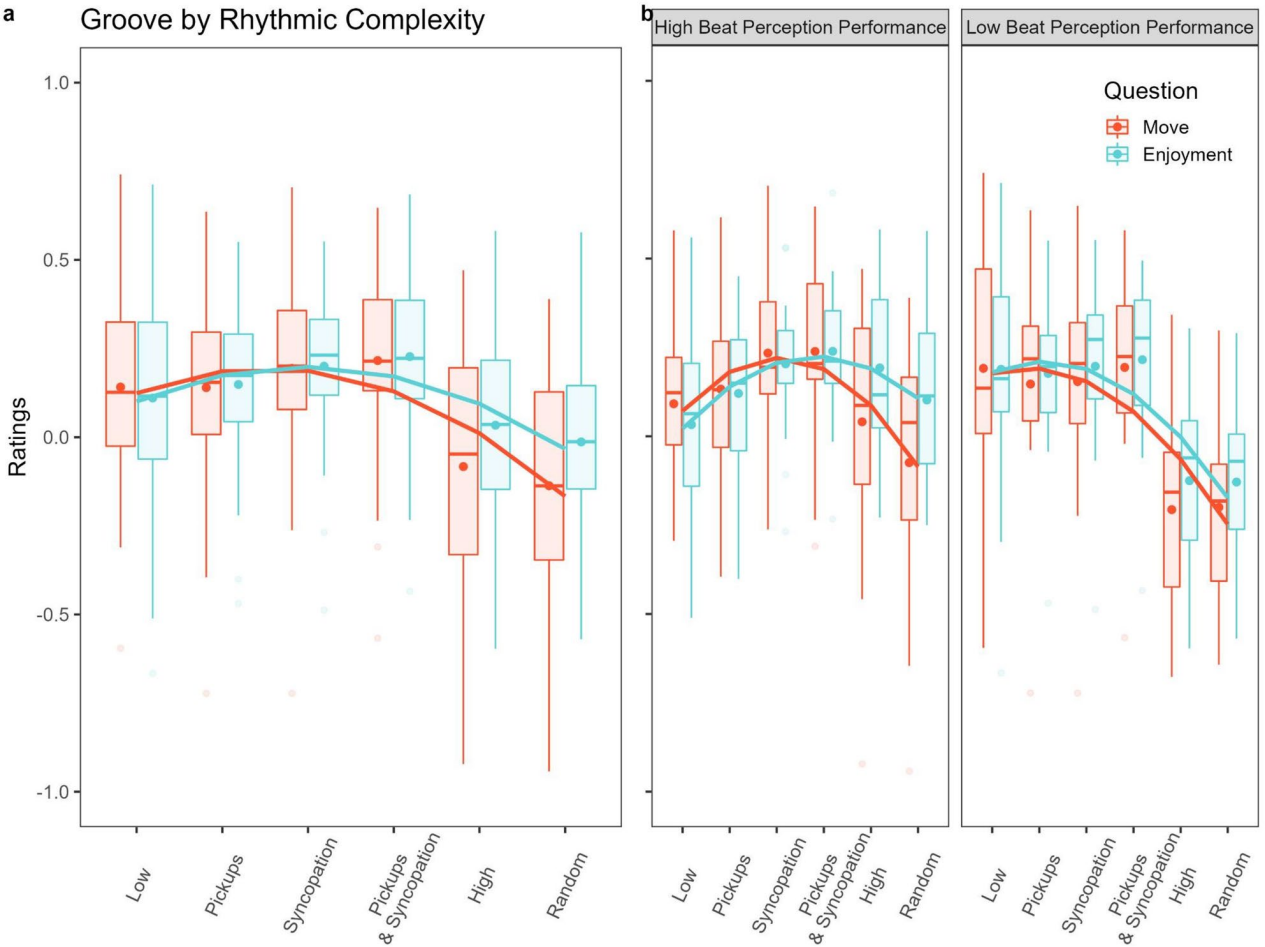
Quadratic models of the behavioral results across Rhythmic Complexity. (**A**) Quadratic models for Urge to Move, Enjoyment, and Perceived Arousal with individual subject predictors. Urge to Move and Enjoyment displayed significant quadratic trends. (**B**) Groove ratings across Rhythmic Complexity split by performance on the CA-BAT. There was a significant interaction between Beat Perception and the linear relationship between Rhythmic Complexity for Enjoyment.

As expected, adding slopes for Rhythmic Complexity improved the model fit for all ratings. However, the quadratic slope significantly improved the fit for Urge to Move (χ^2^(1) = 14.643, p < 0.001) and Enjoyment (χ^2^(1) = 20.774, p < 0.001) while our control question, Perceived Arousal, only trended toward a significantly better fit (χ^2^(1) = 3.429, p = 0.064). Follow-up tests revealed significant negative quadratic (i.e., inverted U-shaped) trends for both Urge to Move (*b*(29) = − 3.167, 95% CI [− 4.643, − 1.691]) and Enjoyment (*b*(29) = − 2.659, 95% CI [− 3.64, − 1.675]), but not Perceived Arousal (*b*(29) = − 0.694, 95% CI [− 1.432, 0.043]). The significant quadratic predictors for Urge to Move and Enjoyment ratings are plotted in Fig. 2a.

Adding Beat Perception group to the mixed effects models yielded similar significant quadratic trends for Urge to Move (*b*(28) = − 3.738, 95% CI [− 5.804, − 1.672]) and Enjoyment (*b*(28) = − 2.664, 95% CI [− 4.055, − 1.27]) that better fit their linear equivalents (Urge to Move: χ^2^(2) = 15.253, p < 0.001; Enjoyment: χ^2^(2) = 20.774, p < 0.001). However, Beat Perception did not significantly impact any ratings except as an interaction with Enjoyment’s linear trend which thus resulted in a slightly better model fit (χ^2^(3) = 9.247, p = 0.02). Follow-up tests revealed this was driven by the Low Beat Perception Performance group exhibiting a significant negative linear trend (*b*(14) = − 3.618, 95% CI [− 6.044, − 1.193]) that was absent in the High Beat Perception Performance group. This indicates that while both High and Low Beat Perception groups showed prominent quadratic trends for both Urge to Move and Enjoyment, only the Low Beat Perception Performance group had a significant linear trend that improved model fit for Enjoyment. This is plotted in Fig. 2b.

The mixed ANOVA comparing High and Low CA-BAT Performance groups’ Urge to Move ratings to the High Complexity and Random drumbeats yielded a marginally significant interaction between the two factors (F(1,28) = 4.492, p = 0.043, η^2^G = 0.022) driven by a small effect in the High Beat Perception Performance group (F(1,14) = 5.189, p = 0.039, η^2^G = 0.077) showing higher ratings for the High Complexity relative to the Random drumbeat that was absent in the Low Beat Perception Performance group (F(1,14) = 0.115, p = 0.740, η^2^G < 0.001). For Enjoyment, a similarly marginal increase in ratings for the High Complexity compared to the Random drumbeat was found for both High and Low Beat Perception Performance groups (F(1,28) = 5.490, p = 0.026, η^2^G = 0.025) alongside a slightly larger group difference where High Performers rated both drumbeats somewhat higher than Low Performers (F(1,28) = 6.108, p = 0.020, η^2^G = 0.160). No significant main effects or interactions were found for Perceived Arousal. Given the inconsistency between Urge to Move and Enjoyment ratings, these results should be taken with some caution.

**Figure 3:**
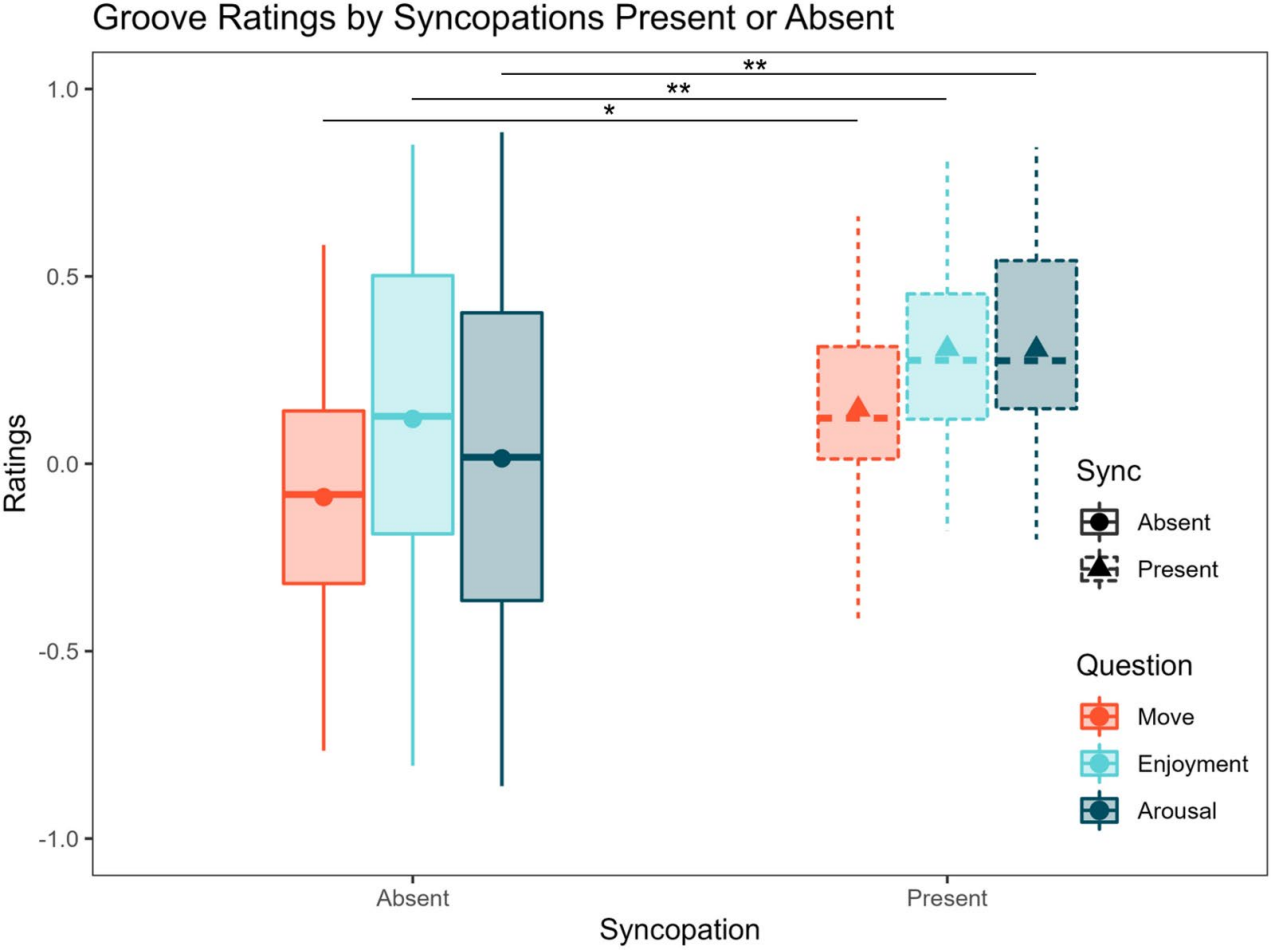
Ratings with pickups (present or absent) and syncopations (present or absent) analyzed orthogonally. The presence of syncopations results in greater ratings of urge to move, enjoyment, and perceived arousal regardless of pickups’ presence. Large dots and triangles represent averages. Single asterisk is p < 0.05, two asterisks p < 0.01.

Using the 2 × 2 design, a two-way repeated measures ANOVAs with within-subjects factors Pickups and Syncopation revealed that Syncopation, but not Pickups, significantly boosted ratings of Urge to Move (F(1,29) = 4.781, p = 0.037, η^2^G = 0.045), Enjoyment (F(1,29) = 10.515, p = 0.003, η^2^G = 0.095), and Perceived Arousal (F(1,29) = 8.665, p = 0.006, η^2^G = 0.085) with no significant interaction between the two factors. These results are depicted in the boxplots in Fig. 3 below.

### Pupillometry results

**Figure 4:**
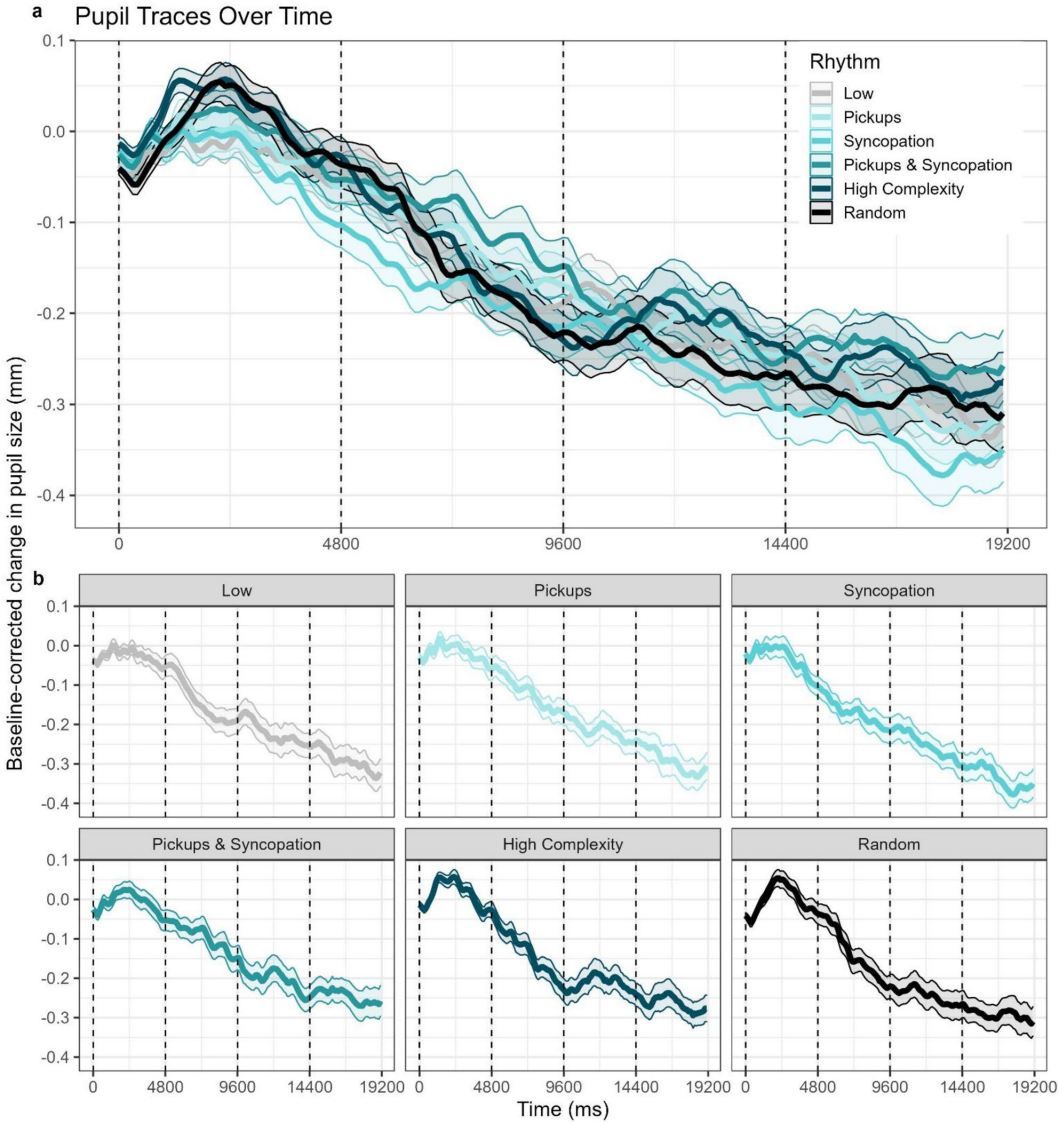
Pupil trace plots for all rhythm conditions over time. Ribbons represent within-subject 95% confidence intervals. Dashed vertical lines represent the boundaries where the first five rhythms looped. (**A**) Pupil traces for all rhythm conditions plotted against each other. (**B**) Pupil traces for each individual rhythm condition plotted separately for better visibility.

Binned and averaged pupil traces of each rhythm with within-subject confidence intervals are plotted in Fig. 4. All conditions demonstrate a sudden dilation consistent with the classic stimulus onset effect in the first repetition out of four, potentially masking effects of interest. To ensure that our drift rate results are untainted by such startle effects, this first repetition window was excluded from further analyses.

**Figure 5:**
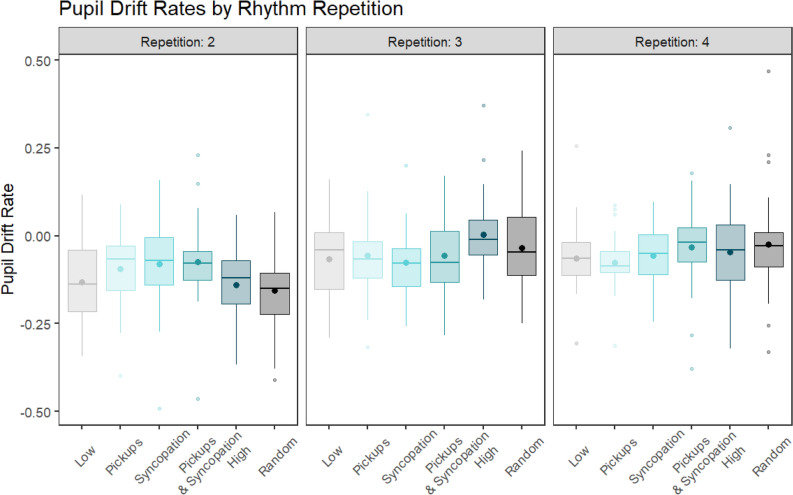
Pupil drift rates by condition across the remaining three within-trial drumbeat repetitions. Drift rates were calculated by averaging the pupil sizes in the first and last three time bins of each repetition with each trial and then computing the slope between these two averages. Large dots represent averages.

**Figure 6:**
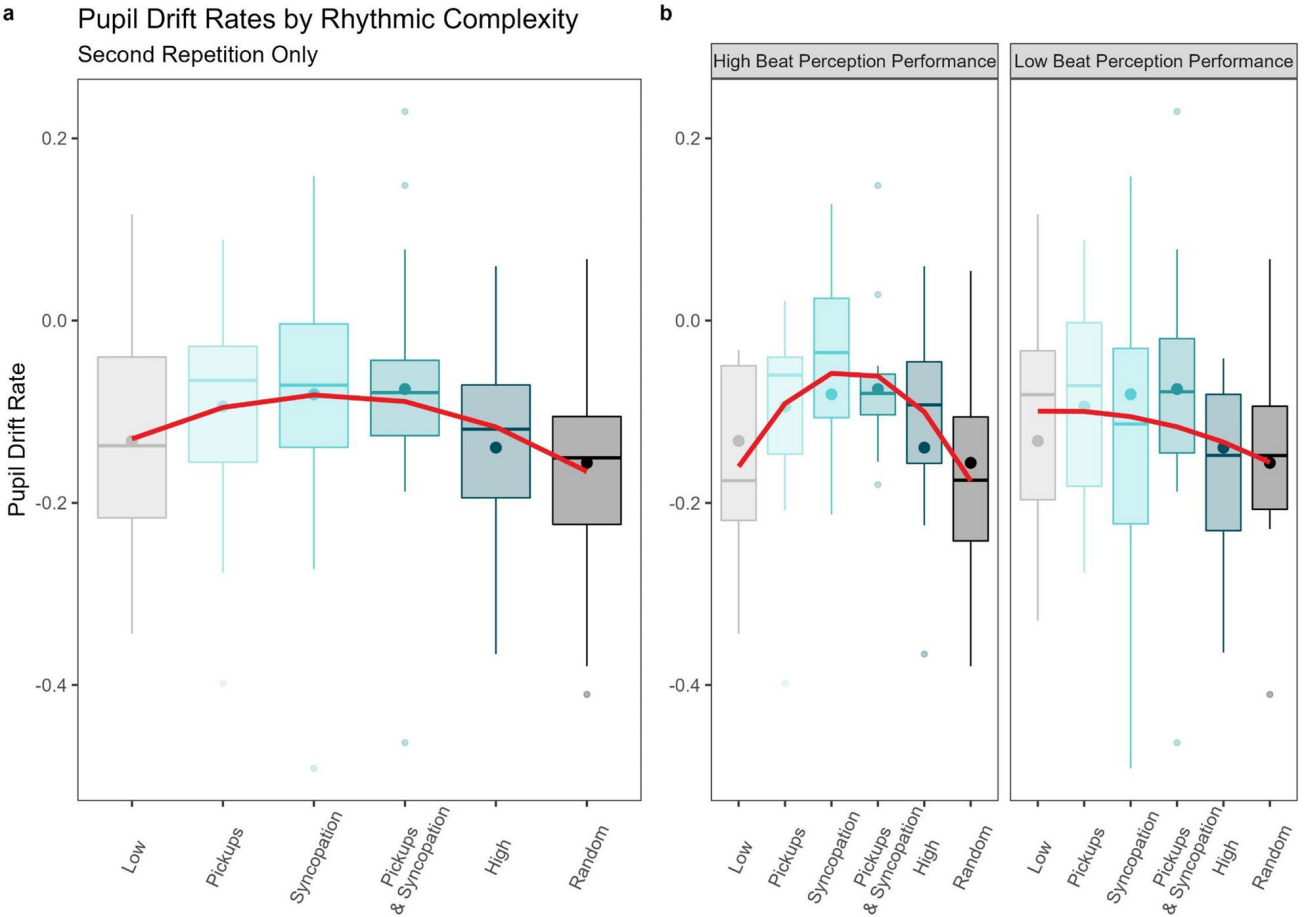
Quadratic fits for the pupil drift rate across rhythmic complexity for Repetition 2 where the significant interaction was found. (**A**) Quadratic predictor of pupil drift rates by rhythm condition during the second repetition of the drum pattern within the trials. Large dots represent averages. (**B**) Pupil drift rates with quadratic predictors by high and low beat perception performance. High performers displayed a significant quadratic relationship with rhythmic complexity while low performers did not.

The pupil size’s drift rate, representing the degree of attentional maintenance or fatigue, is plotted over the remaining three repetitions of the drumbeats in Fig. 5. A repeated measures ANOVA on the pupil drift rate with within-subject factors Rhythm and Repetition revealed a significant modest effect of Repetition (F(1,29) = 26.774, p < 0.001, η^2^G = 0.105) and a smaller but significant interaction between the two factors (F(5,145) = 2.434, p = 0.038, η^2^G = 0.044). Post-hoc tests revealed this interaction to be driven by a main effect of Rhythm found only in the second repetition (F(4.517,130.994) = 3.104, p = 0.0139, η^2^G = 0.067, Huynh–Feldt corrected) and a trend in the third repetition (F(5,145) = 2.009, p = 0.081, η^2^G = 0.054). For this reason and from visual inspection of the entire pupil trace time series, we chose to focus further analyses on the second repetition alone. Remarkably, the pupil’s drift rate during the second repetition mirrors the Urge to Move and Enjoyment ratings in the behavioral portion of the experiment. Using the same mixed effects modeling procedure as the behavioral data**,** we found that a quadratic model fits the pupil drift rate data significantly better than a linear model (χ^2^(1) = 9.721, p = 0.002). Follow-up contrasts showed that this quadratic trend for Rhythm was significant (*b*(148) = − 0.349, 95% CI [− 0.557, − 0.140]), demonstrating an inverted U-shape. This is depicted in Fig. 6a.

Adding beat perception as a fixed effect like we did with the behavioral data improved model fit for the pupil drift rates as well (χ^2^(2) = 15.939, p < 0.001). Follow-up tests revealed a main effect of the quadratic trend (*b*(146) = − 0.605, 95% CI [− 0.890, − 0.321]) and a significant interaction with Beat Perception (*b*(146) = 0.513, 95% CI [0.111, 0.915]). This interaction was driven by a significant quadratic trend that was only present in the High Beat Perception Performance group (*b*(14) = − 0.428, 95% CI [− 0.619, − 0.237]), indicating this group exhibited an inverted U-shape while the Low Beat Perception Performance group did not. This is plotted in Fig. 6b.

**Figure 7:**
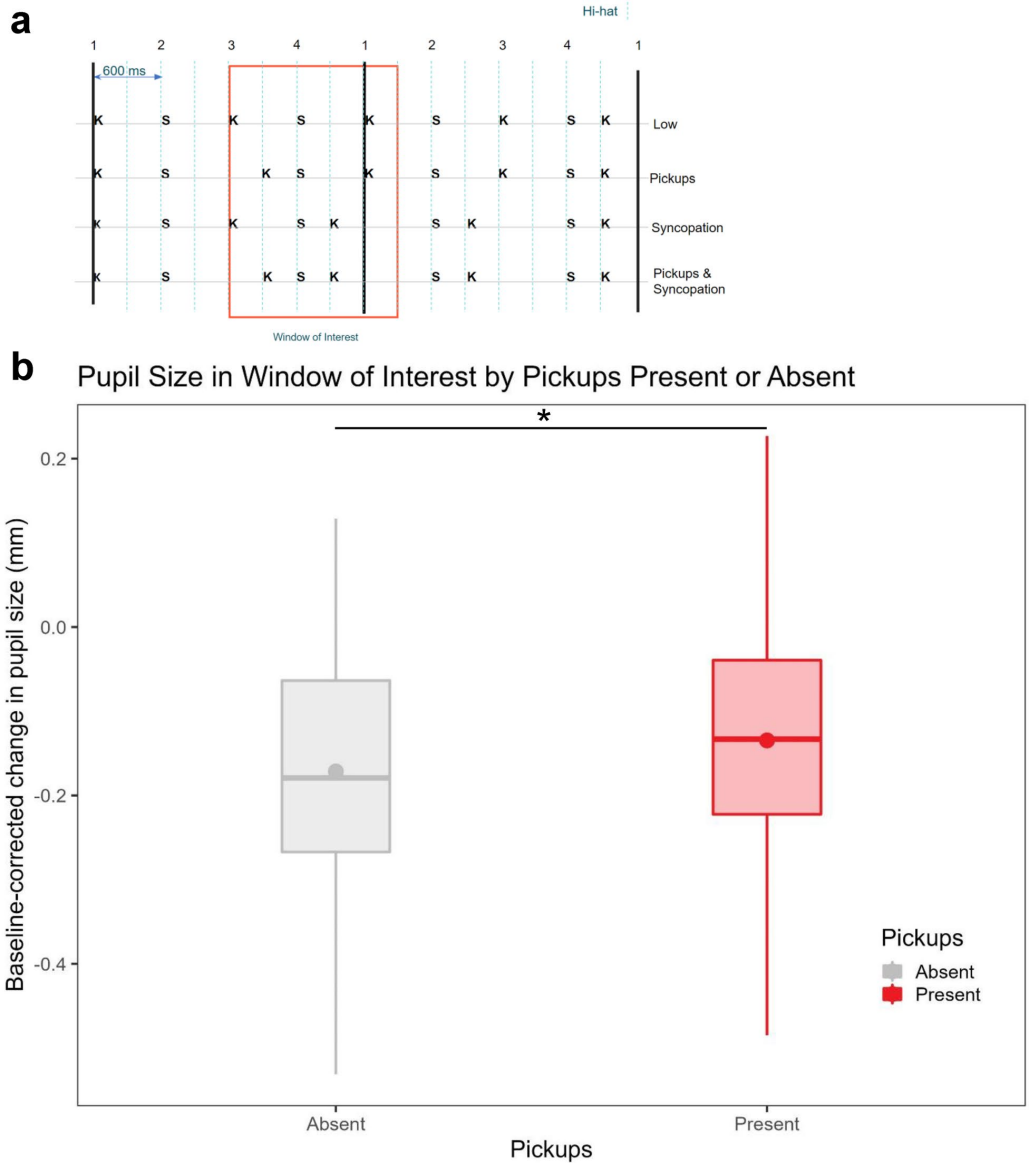
Pupil Size analysis by pickups (Present or Absent) and syncopations (Present or Absent) in the window of interest where the pickup manipulation occurred. (**A**) Window of interest for further analysis. Dashed lines represent hihat hits while Ks represent kick drum hits and Ss represent snare drum hits. Low = Pickups absent, Syncopations Absent, Pickups = Pickups Present, Syncopations Absent, Syncopation = Pickups Absent, Syncopations Present, Pickups and Syncopation = Pickups Present, Syncopations Present. (**B**) Boxplots showing pupil size in the window of interest by Pickups and Syncopation. Large dots and triangles represent group averages.

Next, we repeated our analysis of pickups vs. syncopations using average pupil size data on the entire time window. Here, a repeated measures ANOVA with the factors pickups (Present or Absent) and syncopation (Present or Absent) revealed a significant main effect of pickups (F(1,29) = 4.421, p = 0.044, η^2^G = 0.011). To confirm that this effect was indeed driven by the actual presence of the pickups, we ran this analysis on time windows surrounding the pickups. Given the temporal resolution of the pupil dilation response, we chose 1500 ms windows starting with the standard kick and ending 300 ms after the downbeat of the second bar to ensure each window had the same number of events (two kicks and a snare). This is illustrated in Fig. 7a. The repeated measures ANOVA corroborated this suspicion: there was a significant main effect of Pickups (F(1,29) = 4.626, p = 0.040, η^2^G = 0.013) with no effect of Syncopation or interaction, indicating greater pupil dilations in the two conditions with pickups. This is plotted in Fig. 7b.

## Discussion

In this study, we aimed to investigate pupillometric arousal in the context of groove and its relation to rhythmic complexity using a broad range of rigorously controlled drumbeat stimuli with the novel distinction between pickups and syncopations. We replicated previous behavioral results demonstrating an inverted U-shaped relationship between rhythmic complexity and groove, a relationship that seems less linear with rhythmic expertise as assessed by a beat perception test. We found that rhythms rated groovier were associated with more sustained attention as measured by the pupil size’s drift rate and that this also mapped onto groove ratings split by beat perception ability. Finally, pickups evoked greater pupil dilations while syncopations did not, whereas syncopations resulted in higher groove ratings while pickups exerted no effect on ratings.

### Groove ratings

First and foremost, groove ratings confirmed previous findings^4,5,7,29,34,35^. However, our results also go beyond replication and add nuance by investigating pickups orthogonally to syncopation. While syncopation is certainly one way of manipulating rhythmic expectations, we discovered that their combination with pickups, that is, unstressed notes that reinforce the following strong beat (pulse) or the beginning of a new measure (meter)^31^, is what produced maximal groove in our sample of drumbeats, as was previously hypothesized by Sioros et al.^5,6^. This characterization of groove is in line with both the descriptive musicological model proposed by Sioros et al.^32^ and predictive coding as we will discuss further in the following subsection.

Our analyses using beat perception performance as an individual difference also fit neatly within the predictive coding framework. Qualitatively, High Performers on the CA-BAT displayed inverted-U curves centered closer to the moderate levels of rhythmic complexity whereas ow Performers exhibited a significant negative linear trend that the High Performers did not. At upper levels of complexity, groove ratings are only enhanced by repetition in subjects with high CA-BAT performance, implying that the enjoyable urge to move to rhythms is indeed related to global predictions about their structures should they be perceived. Low Performers also appeared to find the Low Complexity rhythm groovier than high performers, likely because our Low Complexity rhythm was still complex enough to produce prediction errors for them to suppress. The high performance group, however, would not have this experience since their global model of the beat is strong enough to automatically suppress these smaller errors without the need for active inference. This is supported by studies like those of Matthews et al.^39^ where experienced musicians displayed more pronounced quadratic effects in their groove ratings. These findings, however, should be taken with some caution given the inconsistent and marginal nature of our results, especially when comparing ratings of the High Complexity and Random drumbeats between groups. Further, using the entire distribution of CA-BAT scores as a linear predictor in our models was not significant, likely because our sample size was not particularly large or widely dispersed which may have adversely impacted our effect sizes. Thus, more focused work is needed to definitively support these claims.

### Attentional maintenance

Our groove ratings were most closely mirrored by the pupil drift rate, suggesting that more sustained attention is associated with greater groove. This relationship persisted, albeit with similarly small effect sizes, when subjects were split by their CA-BAT performance as well. This is consistent with the hypothesis that an active process of correcting prediction errors with attentional resources underlies the enjoyable urge to move to music. We believe that this better maintenance of attention was the product of the interplay between pickups and syncopations in our stimuli. This drift, however, seems to approach floor with our musical stimuli after around 10 s, indicating that habituation can occur and mask these differences over extended periods of time.

### Divergent/complementary roles for pickups and syncopations

Analyzing groove ratings and pupil size data with syncopations and pickups as separate factors exposed a dissociation where syncopations, but not pickups, significantly boosted ratings but pickups, not syncopations, evoked greater pupil dilations. While this may seem puzzling at first, in the context of our stimuli and the predictive contexts they created together, this can be explained by their different musical functions and the information that they feed to higher-order predictions about the metric structure. Syncopations, by generating prediction errors that challenge global predictions of pulse, create the primary tension that compels us to actively correct them with our movement. Pickups, on the other hand, may strengthen global predictions regarding pulse and meter by immediately fulfilling the expectation that events occur on strong beats, that is, they point out important beats by leading up to and anticipating them, in accordance with the previous hypotheses of Sioros et al.^5,6^. This covert deployment of attentional resources to the pickups occurs regardless of the presence of syncopations and is thus reflected in greater pupil size.

To support the arguments above, we linked pupil dilations to the specific window where the pickups manipulation occurred in our stimuli; this effect endured through all repetitions within the trials. Because the time window’s onset corresponded to 300 ms before the pickup in the Pickups and Pickups and Syncopation rhythms, it seems possible that the pupil may have dilated in anticipation of the beat to strengthen global predictive models of the metric structure. This explanation adds support to the predictive coding literature where the brain is theorized to construct top-down predictions about future sensory experiences that are then used to update those predictions^52,53^.

Pickups are especially intriguing because they might be thought to reinforce the pulse and meter rather than challenge it, and thus compensate for the subversive effect of syncopations which lack the subsequent event on the strong beat^33,54^. In predictive coding terms, both pickups and syncopations may produce local prediction errors since they fall on weak beats (i.e., they violate isochrony), but they propagate different information to higher-order predictions about the metric structure. Because pickups are paired with strong beat events that confirm predictions of the global rhythmic structure, the local prediction errors from the pickups are more precise than those arising from the more unexpected syncopations that lack immediate clarification and consequently call the global rhythmic structure into question. That is, rather than being perceived as unexpected events, pickups’ close proximity to a strong beat event immediately resolves the challenge to isochrony and strengthens the global model whereas this challenge goes unchecked for syncopations. Neurophysiologically, the brain may release norepinephrine to increase the gain of the picked up strong beat and strengthen the metric model, whereas the omission of this strong beat in syncopations needs to be suppressed with movement because it calls the metric model into question. Thus, while syncopations generate the metrical uncertainty that may demand resolution through movement, pickups strengthen the internal model that could be used to guide movements. These movements are then used to reinforce the metric model itself in a feedback loop.

### Limitations and future directions

While we believe our results are consistent with predictive coding, the inverted U-shaped relationship found in the groove ratings and pupil drift rate could potentially be a result of familiarity since most music composed in the Western musical traditions contains moderate amounts of rhythmic complexity (e.g., a mixture of both pickups and syncopations). Predictive coding elegantly posits that music was composed this way because of predictive processes, but it is entirely possible that this occurred for other reasons that then became encultured and familiar. This hypothesis would be consistent with recent evidence presented by Sioros et al.^6^, where algorithmically-generated random syncopation patterns were less effective in evoking groove than the original syncopating patterns of the music excerpts that had a similar degree of syncopation but were created by musicians. We did not assess familiarity in this study because we composed our own stimuli (thus, the participants should not have explicitly recognized any of our drumbeats), but cross-cultural work should be done to disentangle the effects of enculturation and rhythmic complexity.

An alternative explanation for why pickups elicited greater pupil dilations is that the pickups created phenomenal accents on the subsequent strong beat^55^. That is to say, the pickup primed the following beat so that it sounded illusorily louder than other notes, which then evoked a dilation in the pupil. Indeed, past psychoacoustics research has reported small enhancements in perceived loudness of secondary tones with paired sound sequences around the same time interval as our stimuli (300 ms)^56,57^. Since physical loudness differences have been shown to result in greater pupil dilations^58,59^, this account seems plausible and indeed we cannot rule out this possibility with the data presented here. However, because accents direct attention, we take this interpretation to be complementary to our own that pickups cue attention to strong beats to emphasize the metric model in a sort of attentional priming^60^.

Another reason why pickups resulted in greater pupil dilations is potentially because of the number of events. Although we controlled for this as best as we could by ensuring that there were equal kick, snare, and hihat hits in each condition, it is possible that the pupillary response to syncopations occurs at a longer timescale than for pickups. Because syncopations can only be appraised as such after the omitted strong beat has passed, the response for syncopations may have extended beyond our window of interest while the more immediate dilation for pickups was captured. While this confound may have been mitigated by the additional 300 ms after the downbeat in our window of interest (600 ms after the kick in the syncopated conditions), we nevertheless contend that any potential dilation delay is captured by the drift rate analyses.

On a related note, a shortcoming of pupillometry is that the temporal resolution is limited and we cannot directly probe the evoked responses to individual pickups and syncopations. Many researchers have cleverly found ways to remedy this with deconvolution^61–64^. However, as Fink et al.^65^ note, estimating the delay between stimuli and the pupil responses is not always so straightforward and has been shown to differ depending on whether motor responses are required^63^. Temporal alignment may prove even more difficult when using musical stimuli where anticipation changes response latency over time. Moreover, this temporal alignment may also vary with different types of musical anticipation (e.g., for syncopations vs. pickups). Fink and colleagues’ forward modeling method avoids this issue, but the interpretation shifts from evoked pupil responses to fitting predictive models. In order to more directly measure both rhythmic entrainment and quick, evoked responses without introducing theoretical assumptions, we plan to record EEG in further investigations of groove for its greater temporal resolution. In addition to entrainment, event-related potentials to on- and off-beat notes in different rhythmic contexts could elucidate finer differences between pickups and syncopations.

We further plan to extend our behavioral findings regarding the effect of repetition at high levels of rhythmic complexity by beat perception ability to lower levels of complexity. Does repeating a rhythm continue to result in higher groove ratings for only those with strong beat perception abilities or does it generalize to everyone when the beat is easier to perceive? In this way, we can directly modulate global predictions through repetition at every level of metric complexity to disentangle pure predictive processes from musicological ones.

## Conclusions

To our knowledge, this is the first rigorously controlled study of pupil size changes over a broad range of rhythmic complexity that encompasses both pickups and syncopations in order to investigate the neurophysiological correlates of groove. Previous studies either did not fully explore the upper end of complexity or did not clearly distinguish the role of pickups. Here we replicate the canonical inverted U-shaped relationship between rhythmic complexity and groove ratings, including that this effect is enhanced by musical ability using a psychoacoustic test rather than participant demographics. These results seem consistent with the pupil drift rate, suggesting that groovier rhythms hold attention longer than ones rated less groovy. Moreover, we found divergent but complementary effects of syncopations and pickups on groove ratings and pupil size, respectively, extending previous findings by discovering a distinct predictive role for pickups. Specifically, while syncopations may demand our movement to enforce the metric model, pickups evoke greater pupil dilations and cue our attention to strong metric positions without our own movement. This thus lends correlative support to the predictive coding account where groove is envisioned as an embodied resolution of precision-weighted prediction error^8,9^

## Supplementary Information

Supplementary Information.
