# Supplementary material for: Pupil drift rate indexes groove ratings
Source: Sci Rep. 2022 Jul 8;12:11620. doi: 10.1038/s41598-022-15763-w (PMC9270355; doi:10.1038/s41598-022-15763-w)
Supplement: Supplementary file 1 — Supplementary Information. [file 41598_2022_15763_MOESM1_ESM.docx]

**Supplementary Materials
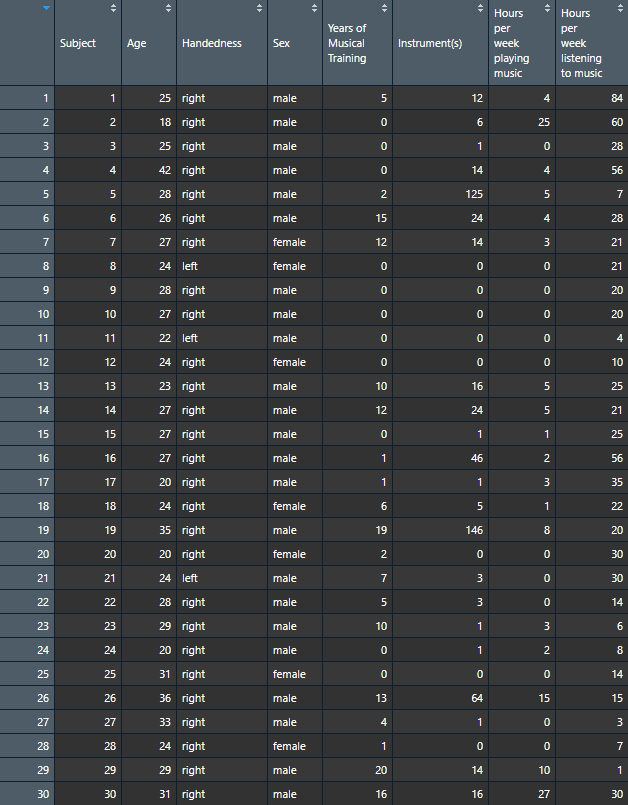
**

**Table 1.** Subject demographics. Key for instrument column: 0=None, 1=Strings, 2=Percussion, 3=Brass, 4=Piano, 5=Voice, 6=Other (including programming).


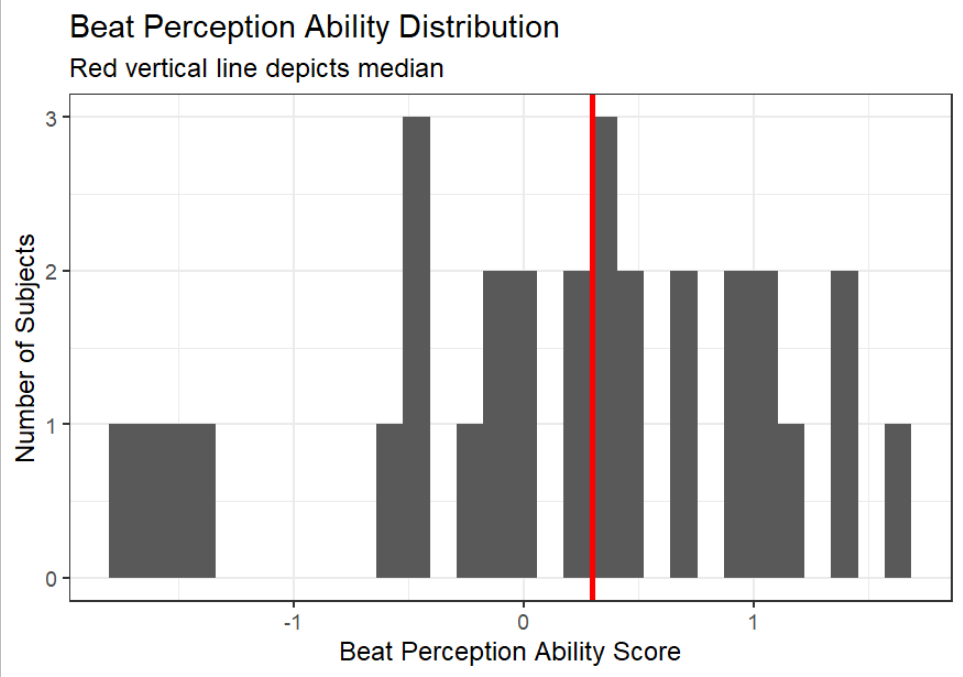


**Supplementary Figure 1.** Histogram of Beat Perception Ability scores obtained with the CA-BAT. The median is marked in red.


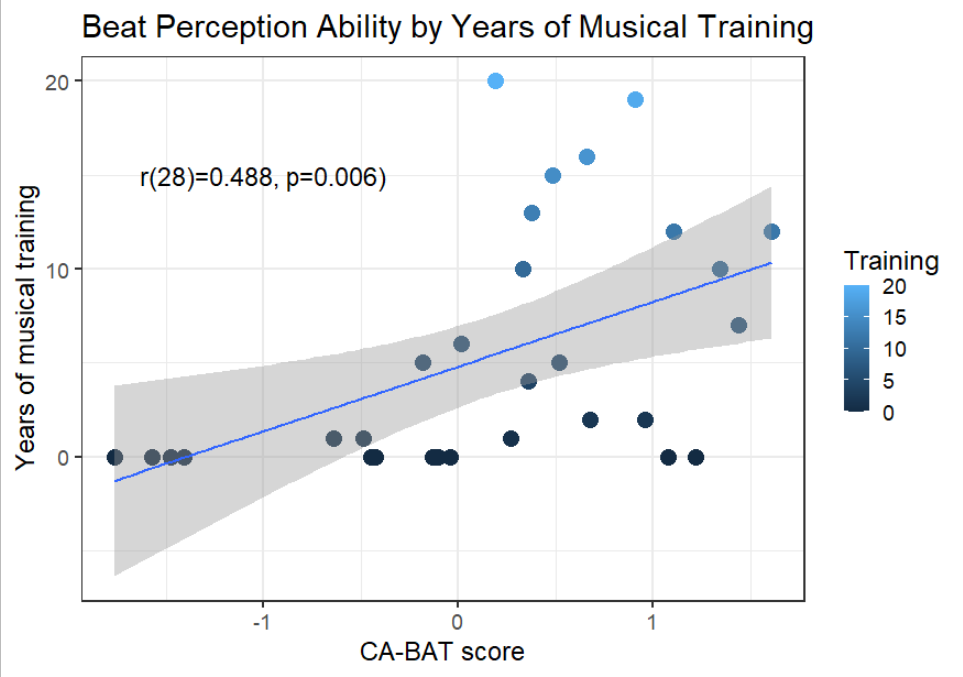


**Supplementary Figure 2.** Positive correlation between Beat Perception Ability and musical training.


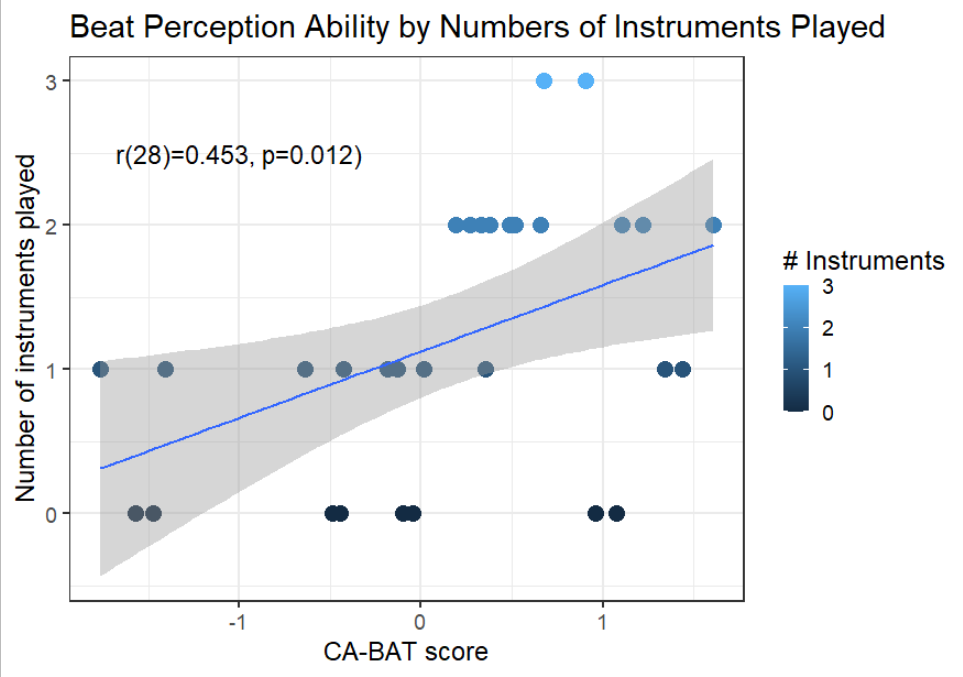


**Supplementary Figure 3.** Correlation between Beat Perception Ability and number of musical instruments played.
